# Innate Immunity of Adipose Tissue in Rodent Models of Local and Systemic* Staphylococcus aureus* Infection

**DOI:** 10.1155/2017/5315602

**Published:** 2017-03-27

**Authors:** Andreas Schmid, Thomas Karrasch, Miriam Thomalla, Jutta Schlegel, Bernd Salzberger, Andreas Schäffler, Frank Hanses

**Affiliations:** ^1^Department of Internal Medicine III, Giessen University Hospital, 35392 Giessen, Germany; ^2^Infectious Diseases Unit, Regensburg University Hospital, 93053 Regensburg, Germany; ^3^Emergency Department, Regensburg University Hospital, 93053 Regensburg, Germany

## Abstract

*Background*. The role of adipose tissue in systemic inflammation during bacterial infection is unclear. Effects of* Staphylococcus aureus* infection on adipocytes in rodent models of experimental endocarditis and peritonitis, the impact of* S. aureus* infection on gene expression in epididymal and subcutaneous adipose tissue, and effects of* S. aureus* infection on the toll-like receptor-2- (TLR2-) cathelicidin pathway in vivo and in vitro were investigated.* Material and methods.* The rat model of catheter-induced* S. aureus* endocarditis and the mouse model of* S. aureus*-induced peritonitis were used for infection experiments, gene expression profiling in adipose tissue, and measurement of cytokines. 3T3-L1 adipocytes were analyzed for expression of the TLR2-cathelicidin pathway.* Results*. Upon systemic bacterial infection by* S. aureus*, there is a shift from anti- to proinflammatory cytokines in serum and in adipose tissue gene expression. The TLR2-cathelicidin pathway is increasingly expressed during adipocyte differentiation in vitro and is induced upon stimulation by synthetic lipopeptides.* Conclusions*. Systemic infection by Gram-positive bacteria induces proinflammatory transformation of adipose tissue sites distinct from infection sites, documented on the levels of gene expression and secreted mediators. The TLR2-cathelicidine pathway is expressed and highly inducible in adipocytes in vitro. Lipopeptides are important immune-modulators of adipocytes in both gene expression and protein secretion.

## 1. Introduction

Besides its role as an endocrine gland with pleiotropic function in whole body metabolism and inflammation [1–7], adipose tissue is increasingly becoming recognized as part of the innate immune system [8–10]. This latter point of view is based on the fact that adipocytes synthesize and secrete numerous components of the innate immune system [10, 11] such as toll-like receptors (TLRs) [10, 12–14], complement factors, C1q/TNF-related proteins (CTRPs) [10, 12, 15, 16], cytokines (e.g., interleukin-6 (IL-6), TNF), chemokines (e.g., MCP-1), and pro- and anti-inflammatory adipokines (e.g., resistin, visfatin, leptin, and adiponectin) as well as antibacterial peptides such as lipocalin-2 and cathelicidin [17]. It could be demonstrated earlier by our group and others [9, 13, 18, 19] that functional toll-like receptors are expressed on adipocytes whose ligand-specific activation modulates cytokine, adipokine, and chemokine release from adipocytes. Thus, adipose tissue is not only linked to metabolic inflammation [20, 21] (“adipoflammation”) and insulin resistance but also linked to local and systemic defense against bacteria, viruses, and microbes.

*Staphylococcus aureus (S. aureus)* is not only a commensal bacterium but also a highly effective pathogen. It represents the most frequently isolated human bacterial pathogen from a range of diseases including blood stream infections, endocarditis, sepsis, pneumonia, arthritis, osteomyelitis, and wound-, skin-, soft tissue-, and foreign body-infections [22]. Challenges in treatment include the severity of life threatening invasive* S. aureus* infections and resistance issues especially among methicillin-resistant* S. aureus* (MRSA) strains. Mortality in patients with* S. aureus* bacteremia remains high [23]. Additionally, invasive* S. aureus* infections are also correlated with metabolic disorders and type 2 diabetes mellitus is associated with a higher risk for and poorer prognosis of* S. aureus* infections [24]. Additionally, obesity is (independent of diabetes) a risk factor for* S. aureus* colonization and infections following surgery [25, 26].

*S. aureus* is able to infect adipocytes in vitro and to survive inside these cells for up to five days in a glucose-dependent manner [27]. Adipocyte infection with* S. aureus* thereby decreased adiponectin and resistin release whereas visfatin, MCP-1, and IL-6 secretion were increased [27].* S. aureus* is also able to attach to and internalize into adipose tissue-derived mesenchymal stem cells [28].

Most recently, Zhang et al. [17] published their data in Science journal showing that subcutaneous adipocytes in the skin release the antimicrobial peptide cathelicidin in response to a subdermal infection with methicillin-resistant* S. aureus *(MRSA) in mice. This mechanism was accompanied by adipocyte proliferation and hypertrophy as a result of increased adipogenesis driven by two transcription factors, peroxisome proliferator-activated receptor-*γ* (PPAR*γ*) and zinc-finger protein 423 (ZFP423). Cathelicidin is produced in human and murine adipocytes also in the absence of infection and is induced by a high-fat diet. Cathelicidin inhibits the growth of* S. aureus*, acts proinflammatorily, and stimulates neutrophils [29]. However, obese and insulin resistant mice carrying missense mutations within the leptin receptor gene are more susceptible to* S. aureus *infections [17]. Similarly, humans suffering from obesity and type 2 diabetes mellitus are under higher risk of skin- and soft tissue-infections by* S. aureus*. This discrepancy [30] might be explained by an interference of the infection-adipogenesis-cathelicidin pathway identified by Zhang et al. [17] with leptin and/or insulin receptor signaling pathway. These data open a new window for molecular research on the crossing point of two superhighways, innate immune system and metabolism [30–33].

Though not yet definitively proven, the recognition of* S. aureus* by adipocytes [30–33] might be mediated by the toll-like receptor-2 (TLR2) signaling pathway [30]. TLR2 has been shown to be expressed in adipocytes [9, 18, 19, 34–36] and to recognize lipopeptides (peptidoglycans) produced by bacteria such as* S. aureus* resulting in enhanced IL-6 expression [18]. Stimulation of murine and human adipocytes with the TLR1/2 ligand Pam3Cys and the TLR2/6 ligand MALP-2 differentially modulated the release of IL-6, IL-8, and MCP-1 [9, 13, 18, 36, 37], whereas resistin was either not affected or even downregulated nonsignificantly by both ligands [9]. It seems important to emphasize that the pattern of cytokine and chemokine release caused by lipopeptides is similar to that observed in experiments using viable staphylococci [27]. In accordance with these observations, TLR2* knockout *mice have a reduced adipose tissue MCP-1 expression and macrophage infiltration [38]. It is currently unclear whether systemic infection by* S. aureus* also alters adipose tissue biology at sites distinct from the respective infection, such as peripheral adipose depots in the intra-abdominal and inguinal compartments.

Therefore, it was the aim of the present study to investigate

- the effect of severe infection by* S. aureus* on adipocyte biology in two rodent models of experimental endocarditis and peritonitis;
- the impact of* S. aureus* infection on cytokine and adipokine expression in epididymal (intra-abdominal) and (inguinal) subcutaneous adipose tissue;
- the potential effect of* S. aureus* infection on the postulated adipose tissue-TLR2-cathelicidin pathway in vivo and in vitro in differentiated adipocytes under basal conditions and upon stimulation with the TLR2-agonistic lipopeptides Pam3Cys and MALP-2.

## 2. Material and Methods

An infective endocarditis model in rats and a mouse peritonitis model by infection with* S. aureus* were characterized. Serum, whole blood, peritoneal fluid, epididymal (intra-abdominal), and inguinal (subcutaneous) adipose tissue were analyzed. All animal experiments were approved by the local government agency (numbers 54-2532.1-05/10 and 54-2532.1-14/10).

### 2.1. Bacteria

*S. aureus* strain Newman was used for endocarditis experiments in the rat model,* S. aureus* strain PS80 (a capsular serotype 8 clinical isolate) was used to induce peritonitis in mice. Both strains were kindly provided by Professor J. C. Lee, Boston, USA. For infection assays,* S. aureus* was grown on tryptic soy agar overnight, resuspended in phosphate-buffered saline (PBS), and adjusted to an optical density (OD) at 650 nm of 0.34. Colony forming units (CFU) were verified by serial plate counts.

### 2.2. Animal Models

#### 2.2.1. Endocarditis Model

The rat model of catheter-induced* S. aureus* endocarditis was described previously by our group and others [39, 40]. Catheterized rats (*n* = 7) were challenged intravenously with ~10^∧^5 CFU* S. aureus* strain Newman 48 h after surgery. Heparinized blood was collected daily from each animal by tail vein puncture and plated quantitatively. Surviving rats were euthanized on day 3 after challenge, the hearts were removed and the correct positioning of the catheter and presence of endocardial vegetation were noted. Epididymal adipose tissue was harvested and was immediately flash frozen in liquid nitrogen.

#### 2.2.2. Peritonitis Model

C57BL/6 mice (*n* = 11) were challenged intraperitoneally (ip) with 5  ×  10^∧^8 CFU* S. aureus* strain PS80 as described previously [41], and the animals were euthanized thereafter at the indicated time points. Heart blood was collected and the peritoneal cavity was lavaged with 5 mL PBS. The* S. aureus* CFU/mL peritoneal fluid and blood were determined by quantitative plate counts. Epididymal and subcutaneous adipose tissues were harvested and immediately flash frozen in liquid nitrogen.

### 2.3. Measurement of Serum Cytokine and Adipokine Concentrations by ELISA

Blood was drawn and serum was prepared following standard procedures. The concentrations of resistin, adiponectin, and visfatin in rat and mouse sera were measured by ELISA. Resistin (mouse), leptin (mouse), IL-6 (mouse, rat), and adiponectin (mouse, rat) sandwich ELISA detection systems were purchased from R&D Systems, Wiesbaden, Germany (DuoSet® ELISA development systems). Visfatin (Nampt)-ELISA Kit (mouse/rat) was a product from AdipoGen (distributed from Biomol, Hamburg, Germany).

Each sample was measured in duplicate by ELISA. Values are depicted as means ± double standard error of the mean (2x SEM).

### 2.4. Murine 3T3-L1 Cell Culture

3T3-L1 preadipocytes [42] were cultured at 37°C and 5% CO_2_ in DMEM (Dulbecco's Modified Eagle Medium, Biochrom AG, Berlin, Germany) supplemented with 10% newborn calf serum (NCS, Sigma-Aldrich, Deisenhofen, Germany) and 1% penicillin/streptomycin (PAN, Aidenbach, Germany). Cells were differentiated into adipocytes at confluence by DMEM/F12/glutamate medium (Lonza, Basel, Switzerland) supplemented with 20 *μ*M 3-isobutyl-methyl-xanthine (Serva, Heidelberg, Germany), 1 *μ*M corticosterone, 100 nM insulin, 200 *μ*M ascorbate, 2 *μ*g/mL transferrin, 5% NCS, 1 *μ*M biotin, 17 *μ*M pantothenate, and 1% penicillin/streptomycin (all from Sigma-Aldrich, Deisenhofen, Germany), and 300 *μ*g/mL Pedersen-fetuin (MP Biomedicals, Illkirch, France) [43, 44] for 9 days using a slightly modified protocol as reported in the literature [42, 45–48]. Phenotype was controlled by light-microscopy (appearance of extensive accumulation of lipid droplets). Mature adipocytes at day 9 of differentiation were used for stimulation experiments. Cells were washed with PBS and incubated under serum-free culture conditions. The lipopeptides and TLR2 agonists MALP-2 and Pam3Cys were purchased from Axxora (Loerrach, Germany) and were dissolved in sterile water (from Sigma-Aldrich, Deisenhofen, Germany). Both undifferentiated 3T3-L1 fibroblasts and mature adipocytes were treated with MALP-2 (doses: 50 and 100 ng/mL) and Pam3Cys (50 and 100 ng/mL) and were incubated for 18 h each. Equivalent volumes of water were used as negative controls, and the absence of cytotoxic effects by the stimulation doses applied was approved by measurement of lactate dehydrogenase (LDH) concentrations in cell supernatants (Cytotoxicity Detection Kit, Roche, Mannheim, Germany). After incubation, cell supernatants were taken and frozen at −20°C for further analysis. Cells either were harvested in phosphate-buffered saline and were then undergoing sonification-assisted cell lysis in RIPA (radioimmunoprecipitation assay) buffer in order to gain protein lysates or were further processed for RNA isolation. In case of cellular protein preparations, BCA Protein Assay Kit (Thermo Fisher Scientific, Dreieich, Germany) was applied for measurement of protein concentrations.

### 2.5. Measurement of Cytokine and Adipokine Concentrations in 3T3-L1 Cell Supernatants

The concentrations of resistin, adiponectin, lipocalin-2, MCP-1, and IL-6 were measured by ELISA. Sandwich ELISA detection systems were purchased from R&D Systems, Wiesbaden, Germany (DuoSet ELISA Development Systems). Each sample was measured in duplicate by ELISA. Values are depicted as means ± 2x SEM.

### 2.6. Gene Expression Analysis

The mRNA expression in adipose tissue and in 3T3-L1 fibroblasts and adipocytes was investigated by quantitative real-time PCR. Total RNA was isolated from fat tissue using TRIzol®-Reagent (Life Technologies GmbH, Darmstadt, Germany) in combination with gentleMACS Dissociator and M-tubes (Miltenyi Biotec GmbH, Bergisch Gladbach, Germany) for dissociation. From prepared tissue and harvested 3T3-L1 cells, RNA was isolated using RNeasy® Mini Kit (Qiagen, Hilden, Germany) including DNase digestion (RNase-Free DNase Set, Qiagen). A 1 *μ*g sample of total RNA was reverse transcribed by QuantiTect® Reverse Transcription Kit (Qiagen, Hilden, Germany), if samples were used for single quantitative real-time PCR assays (LightCycler®, Roche Applied Science, Mannheim, Germany, and iTaq™ Universal SYBR® Green, Bio-Rad Laboratories, Munich, Germany). RT^2^ Profiler PCR Arrays (Qiagen, Hilden, Germany) were performed for simultaneous quantification of gene expression in rat adipose tissue. In these experiments, cDNA was synthesized using RT^2^ First-Strand Kit (Qiagen, Hilden, Germany) with an amount of 400 ng total RNA. For single real-time PCR reactions, intron-spanning primers (Metabion, Planegg-Martinsried, Germany) were used specifically for mouse adiponectin (universe: 5′-AGGGAGAGAAAGGAGATGCAG-3′, reverse: 5′-CAGACTTGGGCTCCCACCTC-3′), mouse adiponectin receptor type 1 (AdipoR1) (universe: 5′-AGGCCTGTCCACCATCAC-3′, reverse: 5′-CAGAAGGAGCCCCATTGC-3′), mouse resistin (universe: 5′-TGCTAAGTCCTCTGCCACGTA-3′, reverse: 5′-TCAACTGACCGACATCAGGA-3′), mouse visfatin (universe: 5′-TTTTATGGGTTGCAGTACATTCTT-3′, reverse: 5′-AATGAGCAGATGCCCCTATG-3′), mouse IL-6 (universe: 5′-TTCCATCCAGTTGCCTTCTT-3′, reverse: 5′-TTCTGCAAGTGCATCATCGT-3′), mouse *β*-actin (universe: 5′-TGGAATCCTGTGGCATCCATG-3′, reverse: 5′-TAAAACGCAGCTCAGTAACAG-3′), mouse 18S rRNA (universe: 5′-GATTGATAGCTCTTTCTCGATTCC-3′, reverse: 5′-CATCTAAGGGCATCACAGACC-3′), mouse glyceraldehyde 3-phosphate dehydrogenase (GAPDH) (universe: 5′-TGTCCGTCGTGGATCTGAC-3′, reverse: 5′-AGGGAGATGCTCAGTGTTGG-3′), mouse lipocalin-2 (universe: 5′-ACGGACTACAACCAGTTCGC-3′, reverse: 5′-GGTGGGGACAGAGAAGATGA-3′), mouse uncoupling protein-1 (UCP-1) (universe: 5′-GGGCCCTTGTAAACAACAAA-3′, reverse: 5′-GTCGGTCCTTCCTTGGTGTA-3′), mouse PPAR*γ* (universe: 5′-TTATAGCTGTCATTATTCTCAGTGGAG-3′, reverse: 5′-GGGTGGGACTTTCCTGCTA-3′), mouse T-box1 (Tbx1) (universe: 5′-GGCAGGCAGACGAATGTTC-3′, reverse: 5′-TTGTCATCTACGGGCACAAAG-3′), mouse TMEM26 (universe: 5′-ACCCTGTCATCCCACAGAG-3′, reverse: 5′-TGTTTGGTGGAGTCCTAAGGTC-3′), mouse CD137 (universe: 5′-CGTGCAGAACTCCTGTGATAAC-3′, reverse: 5′-GTCCACCTATGCTGGAGAAGG-3′), mouse TLR2 (universe: 5′-AAACCTCAGACAAAGCGTCAA-3′, reverse: 5′-TTCATGGCTGCTGTGAGTCC-3′), mouse cathelicidin (universe: 5′-CCCAAGTCTGTGAGGTTCCG-3′, reverse: 5′-GTGCACCAGGCTCGTTACA-3′), rat glyceraldehyde 3-phosphate dehydrogenase (GAPDH) (universe: 5′-TACCAGGGCTGCCTTCTCTTG-3′, reverse: 5′-GGATCTCGCTCCTGGAAGATG-3′), rat *β*-actin (universe: 5′-GATCAAGATCATTGCTCCTCCTG-3′, reverse: 5′-AGGGTGTAAAACGCAGCTCA-3′), rat cathelicidin (universe: 5′-CAACCAGCAGTCTTTGGACA-3′, reverse: 5′-TAACTGCTGTGATGCCTTGC-3′), rat TLR2 (universe: 5′-AGCTGGAGAACTCTGACCCA-3′, reverse: 5′-CAAAGAGCCTGAAGTGGGAG-3′), rat PPAR*γ* (universe: 5′-TTATAGCTGTCATTATTCTCAGTGGAG-3′, reverse: 5′-CTGGAGCAGAGGGTGAAGG-3′), rat UCP-1 (universe: 5′-TCGGTACCCACATCAGGCAA-3′, reverse: 5′-CTGGCCTTCACCTTGGATCTGAA-3′), rat Tbx1 (universe: 5′-CAGAATCACCGGATCACGCA-3′, reverse: 5′-AGCGAGCAAAGGCACTTACA-3′), rat CD137 (universe: 5′-CAGGGGTTCTGAGTTCCAGC-3′, reverse: 5′-AGAAAGTCCCAGCCTCACAG-3′), and rat transmembrane protein 26 (Tmem26) (universe: 5′-TTGAACTGGCCAGAGGCTTT-3′, reverse: 5′-CGTCCCCACGAACATCAGAA-3′).

The standard curve method was used for quantification of the results obtained by real-time PCR. Expression of mRNA of investigated genes in each sample was normalized to the mRNA expression level of a housekeeping gene (GAPDH, 18S rRNA or *β*-actin) by calculation of ΔCt. Expression differences between groups were calculated and depicted as ΔΔCt or as fold-expression in comparison to relative control group.

### 2.7. Statistics

Data are expressed as mean values ± 2x SEM. Differences between means were tested for significance using the Mann–Whitney* U *test. One-way ANOVA was used to test for statistically significant differences over time, and two-way ANOVA was used to compare two different groups over time. A *p* value below 0.05 was considered to be statistically significant. For graphical illustration of cell culture experiments, box plot diagrams are shown.

## 3. Results 

### 3.1. Systemic Infection with* S. aureus* and Natural Disease Course of the Fatal Endocarditis Model in Rats

**Figure 1:**
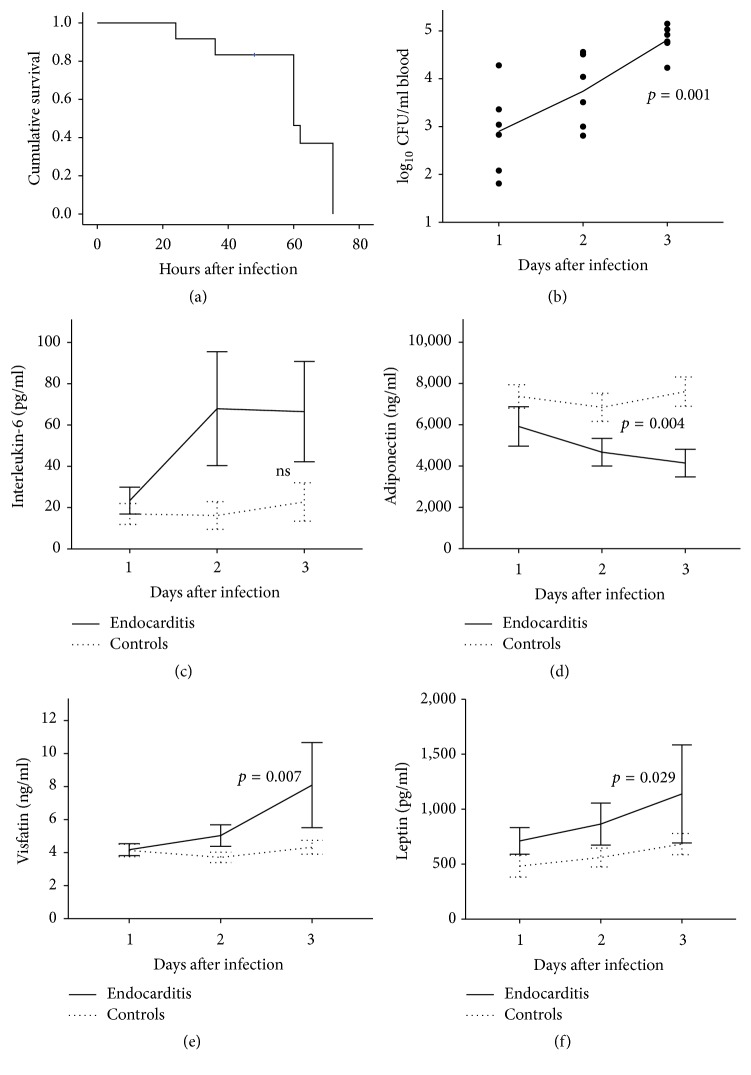
*Natural course of fatal S. aureus endocarditis and serum adipokine concentrations in an experimental rat model.* Serum and whole blood were collected from rats (*n* = 5–7 per time point) suffering from an ultimately fatal* S. aureus* endocarditis. Serum cytokine and adipokine levels were measured by ELISA in duplicate. (a)* Cumulative survival of animals during the natural disease course.* Cumulative survival is given in percent (%) during the time after infection given in hours (h). (b)* Bacteremia during the natural disease course.* Colony forming units are given in units/mL blood by using a logarithmic scale and were documented at day 1, day 2, and day 3 after infection. Bacteremia increased significantly (*p* = 0.001, one-way ANOVA) over time. (c)* Time course of serum IL-6 concentrations in infected (black line) and noninfected control (spotted line) animals.* IL-6 concentrations were increased in* S. aureus* infected rats; however, this trend did not reach significance (ns, not significant). (d)* Time course of serum adiponectin concentrations in infected (black line) and noninfected control (spotted line) animals.* Adiponectin concentrations were significantly (*p* = 0.004, two-way ANOVA) lower in rats with* S. aureus* endocarditis than in uninfected control animals. (e)* Time course of serum visfatin concentrations in infected (black line) and noninfected control (spotted line) animals.* Visfatin concentrations were significantly higher in* S. aureus* infected animals (*p* = 0.007, two-way ANOVA). (f)* Time course of serum leptin concentrations in infected (black line) and noninfected control (spotted line) animals.* Leptin concentrations were significantly higher in* S. aureus* infected animals (*p* = 0.029, two-way ANOVA).

We used the rat endocarditis model as a model for severe and systemic* S. aureus* infection. Infection in this model is ultimately fatal (Figure 1(a)) and rats were sacrificed after 72 h or whenever they appeared gravely ill. The average weight loss 48 h after infection was 8.2%  ± 1.1% of the original body weight. Throughout the course of the experiments, the systemic spread of infection was reflected by continuously increasing levels of bacteremia (Figure 1(b), *p* = 0.001 for trend).

### 3.2. *S. aureus* Endocarditis Alters the Systemic Cytokine and Adipokine Profile in Rats

Although we observed ~3-fold higher levels of the proinflammatory cytokine IL-6 in animals with endocarditis when compared to control animals, these differences did not reach statistical significance due to considerable variability of serum concentrations between animals within the subgroup of* S. aureus* infected rats (Figure 1(c)). In contrast, systemic levels of the anti-inflammatory adipokine adiponectin significantly decreased in serum from rats with endocarditis when compared to control animals (4670.5 ± 669.3 ng/mL versus 6844.3 ± 680.3 ng/mL at day 2 after infection and 4146.0 ± 669.5 ng/mL versus 7604.5 ± 709.7 ng/mL at day 3 after infection; *p* = 0.004 in two-way ANOVA) (Figure 1(d)). In contrast to the anti-inflammatory adiponectin, the proinflammatory adipokines visfatin and leptin increased significantly. Visfatin concentrations (Figure 1(e)) were 5.03 ± 0.65 ng/mL versus 3.71 ± 0.31 ng/mL at day 2 after infection and 8.09 ± 2.59 ng/mL versus 4.33 ± 0.42 ng/mL at day 3 after infection (*p* = 0.007 in two-way ANOVA). Leptin concentrations (Figure 1(f)) were 864.9 ± 191.0 pg/mL versus 560.7  ±  86.0 pg/mL at day 2 after infection and 1138.6 ± 446.1 pg/mL versus 682.6 ± 96.3 pg/mL at day 3 after infection (*p* = 0.029 in two-way ANOVA).

### 3.3. Natural Disease Course of the Nonfatal* S. aureus* Peritonitis Model in Mice

**Figure 2:**
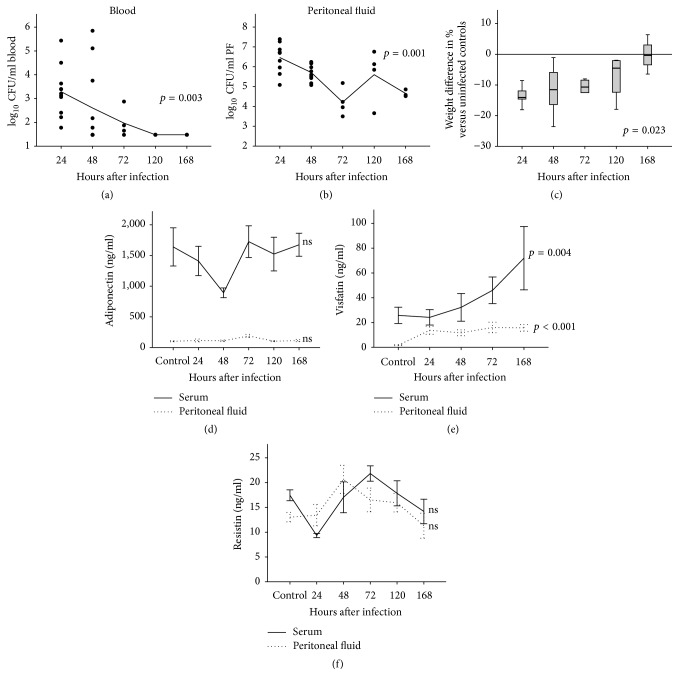
*Natural course of nonfatal S. aureus peritonitis and serum/peritoneal fluid adipokine concentrations in an experimental mouse model.* Serum, whole blood, and peritoneal fluid were collected from C57BL/6 mice with* S. aureus*-induced peritonitis. Serum and peritoneal fluid adipokine concentrations were measured by ELISA in duplicate in *n* = 4–11 animals per time point. (a)* Systemic bacteremia during the natural disease course.* Colony forming units are given in units/mL blood by using a logarithmic scale and were documented from 24 h up to 168 h after infection.* S. aureus* infected mice cleared the initial bacteremia significantly (*p* = 0.003, one-way ANOVA) within 5 days (120 h) after infection. (b)* Peritoneal bacteremia during the natural disease course.* Colony forming units are given in units/mL peritoneal fluid (PF) by using a logarithmic scale and were documented from 24 h up to 168 h after infection. Although viable bacteria were still present in the peritoneal cavity at the end of the experiments after 168 h, the bacterial burden decreased significantly (*p* = 0.001; one-way ANOVA). (c)* Weight loss and weight recovery during the natural disease course.* During the early course of infection, a significant weight loss of 13.4% occurred. This weight loss completely recovered parallel to the decreasing systemic and local bacterial burden. The changes of weight were statistically significant (*p* = 0.023; one-way ANOVA) despite the relatively high variance. (d)* Time course of serum (black line) and peritoneal fluid (spotted line) adiponectin concentrations.* Although an initial trend towards lower serum adiponectin levels after 48 h was observed, adiponectin levels were not significantly (ns) altered in peritoneal fluid and serum during the time course of the experiments. (e)* Time course of serum (black line) and peritoneal fluid (spotted line) visfatin concentrations.* Visfatin levels in serum (*p* = 0.004) and peritoneal fluid (*p* < 0.001) were significantly elevated during the course of the infection. (f)* Time course of serum (black line) and peritoneal fluid (spotted line) resistin concentrations.* Resistin serum and peritoneal fluid concentrations remained unchanged during infection. Although not significantly influenced by infection, resistin was the only adipokine that was detectable with similar concentrations in serum and peritoneal fluid (oscillating between 10 and 20 ng/mL).

We used the murine peritonitis model as an experimental model for a nonfatal* S. aureus* infection. In contrast to the endocarditis model, mice infected intraperitoneally with* S. aureus* cleared the systemic spread of infection after considerable bacteremia after 5 days of infection (Figure 2(a), *p* = 0.003 for trend). Although viable* S. aureus* were still present in the peritoneal cavity 7 days after infection, the bacterial burden gradually declined during the course of the experiments (Figure 2(b), *p* = 0.001 for trend). The resolving infection was reflected by the observation that* S. aureus* infected mice recovered their initial weight loss (−13.4%  ±  0.81% 24 h after infection) within 7 days (Figure 2(c), *p* = 0.023 for trend).

### 3.4. Nonfatal* S. aureus* Peritonitis Modulates Serum and Peritoneal Fluid Adipokine Concentrations Differentially

In contrast to the endocarditis model we found no significant changes in adiponectin serum levels in* S. aureus* infected mice (Figure 2(d)). The initial decline at 48 h did not reach statistical significance. Peritoneal adiponectin concentrations also remained unchanged (Figure 2(d)). Visfatin levels, however, were increased over time in both infection models. In the mouse peritonitis model, visfatin serum levels were 71.9 ± 25.6 ng/mL after 168 h compared to 24.2 ± 6.2 ng/mL after 24 h of infection and 25.8 ± 6.6 ng/mL in uninfected controls (Figure 2(e), *p* = 0.004 in one-way ANOVA). We also found increased visfatin levels in peritoneal fluid of* S. aureus* infected mice (*p* < 0.001 for trend), albeit at a lower level. Resistin was the only adipokine found with similar concentrations in serum and peritoneal fluid of* S. aureus* infected mice (Figure 2(f)). There was no visible and significant trend of resistin levels during the course of infection in both compartments.

### 3.5. Systemic and Local* S. aureus* Infection Alters Gene Expression Levels in Adipose Tissue Depots Distinct from the Sites of Infection

Infection with* S. aureus* altered expression levels of several genes investigated. Genes that showed at least a 2-fold change in the initial screen or whose expression levels were significantly altered are summarized in Table 1 regarding the endocarditis rat model and in Table 2 regarding the peritonitis mouse model.

Genes expressed significantly stronger in epididymal adipose tissue from rats with* S. aureus* endocarditis (Table 1) compared to uninfected animals were the genes for PYCARD (PYD and CARD domain-containing), resistin, and protein tyrosine phosphatase-1 (Ptpn1). Genes expressed significantly lower in epididymal adipose tissue obtained from rats with endocarditis (Table 1) were the genes for the adiponectin receptor type 1 (AdipoR1) and phosphoenolpyruvate carboxykinase-1 (PEPCK-1). Although there was a trend towards lower PPAR*γ* expression, this trend did not reach a statistically significant level. The expression of the adipokines visfatin, leptin, and adiponectin was not different between the groups of experimental animals (Table 1). The gene expression of markers specifically indicating induction of brown adipocyte differentiation (“browning”) was investigated and was completely negative for the marker genes UCP-1, TMEM26, CD136, and TBX1 (Table 1).

In the peritonitis mouse model (Table 2), PYCARD, IL-6, and lectin-like oxidized-LDL receptor-1 (LOX-1) were significantly upregulated in epididymal and in subcutaneous adipose tissue at all time points, whereas MCP-1 was induced only in epididymal but not in subcutaneous adipose tissue. In contrast, resistin, leptin, and PPAR*γ* were downregulated in epididymal but not in subcutaneous adipose tissue (Table 2). Adiponectin, AdipoR1, and visfatin gene expression were not significantly altered (Table 2). Gene expression of markers specifically indicating induction of brown adipocyte differentiation (“browning”) was investigated and there was no clear and consistent induction of the marker genes UCP-1 and CD137 (Table 2).

### 3.6. Expression of TLR2 in Adipose Tissues Samples of Animals Infected with* S. aureus*

Since staphylococci and their lipopeptides are being recognized by TLR2, we investigated the expression of TLR2 on mRNA level (Tables 1 and 2) in the two murine infection models. TLR2 gene expression remained unchanged in rat epididymal adipose tissue and in mouse adipose tissues when compared to control animals.

### 3.7. Expression of TLR2 and Cathelicidin mRNA during 3T3-L1 Adipocyte Differentiation

**Figure 3:**
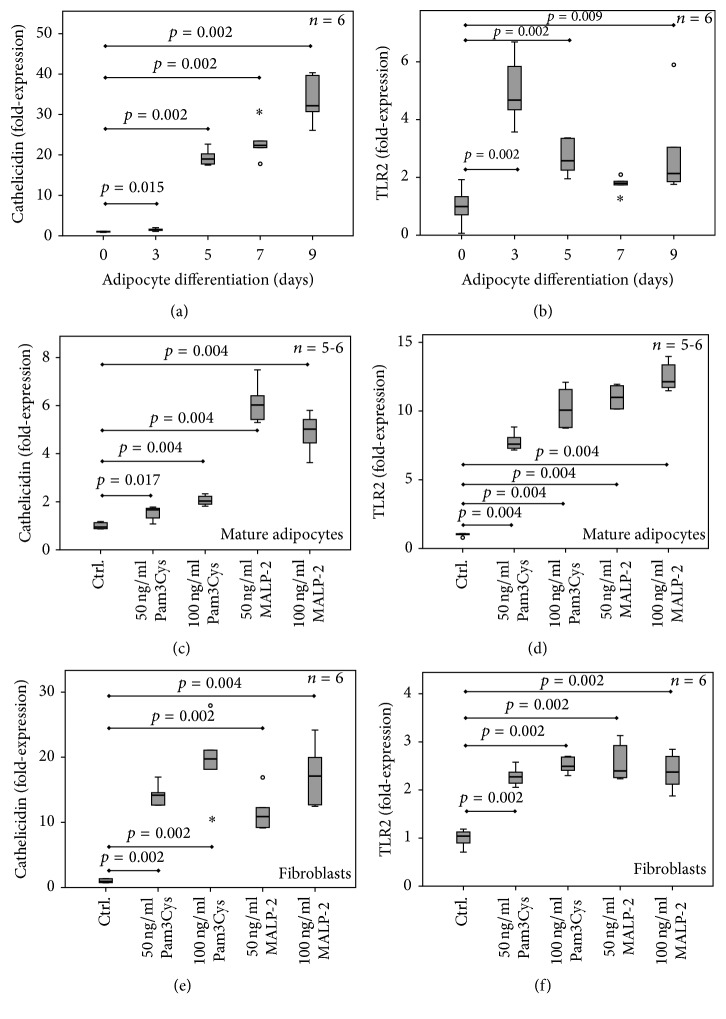
*Expression and regulation of cathelicidin and TLR2 during adipocyte differentiation and upon stimulation with the TLR2 agonistic lipopeptides Pam3Cys and MALP-2*. 3T3-L1 fibroblasts were differentiated into mature adipocytes by applying a hormonal differentiation program. Gene expression analysis was performed during adipocyte differentiation at day 0 (fibroblasts), day 3, day 5, day 7, and day 9 (mature adipocytes). *n* = 4–6 wells were analyzed per group. Box plots are shown indicating the median value and upper and lower quartiles, whiskers (1.5-fold of interquartile range). Outliers are marked by circles and asterisks. TLR2, toll-like receptor-2; Pam3Cys, (*S*)-(2,3-bis(palmitoyloxy)-(2*RS*)-propyl)-*N*-palmitoyl-(*R*)-Cys-(*S*)-Ser(*S*)-Lys4-OH-trihydrochloride (TLR1/2-agonist); MALP-2, macrophage-activating lipopeptide-2 (TLR2/6-agonist). (a)* Cathelicidin gene expression during 3T3-L1 adipocyte differentiation.* Cathelicidin mRNA expression is given as fold-expression in relation to undifferentiated fibroblasts at day 0 of differentiation. (b)* TLR2 gene expression during 3T3-L1 adipocyte differentiation.* TLR2 mRNA expression is given as fold-expression in relation to undifferentiated fibroblasts at day 0 of differentiation. (c)* Cathelicidin gene expression in mature 3T3-L1 adipocytes upon stimulation with the lipopeptides Pam3Cys and MALP-2.* Cathelicidin mRNA expression is given as fold-expression in relation to unstimulated control cells. (d)* TLR2 gene expression in mature 3T3-L1 adipocytes upon stimulation with the lipopeptides Pam3Cys and MALP-2*. TLR2 mRNA expression is given as fold-expression in relation to unstimulated control cells. (e)* Cathelicidin gene expression in 3T3-L1 fibroblasts upon stimulation with the lipopeptides Pam3Cys and MALP-2*. Cathelicidin mRNA expression is given as fold-expression in relation to unstimulated control cells. (f)* TLR2 gene expression in 3T3-L1 fibroblasts upon stimulation with the lipopeptides Pam3Cys and MALP-2*. TLR2 mRNA expression is given as fold-expression in relation to unstimulated control cells. “°” and “*∗*” indicate outliers in the respective data sets displayed as box plots.

We studied expression of TLR2 and cathelicidin mRNA in the 3T3-L1 fibroblast cell line before and after induction of hormonal differentiation (day 0 to day 9) into mature adipocytes. As shown in Figure 3(a), cathelicidin expression is very weak in 3T3-L1 fibroblasts but strongly (*p* = 0.002) induced during adipocyte differentiation in a stepwise manner with highest levels in mature adipocytes at day 9 of differentiation.

As depicted in Figure 3(b), the expression of TLR2 is also low in fibroblasts but is induced early during adipocyte differentiation with a maximum at day 3 (*p* = 0.002) and remains at higher levels in mature adipocytes at day 9 when compared to fibroblasts (*p* = 0.009).

### 3.8. TLR2 and Cathelicidin Gene Expression in Mature Adipocytes and Fibroblasts upon Stimulation with the Lipopeptides MALP-2 and Pam3Cys

In mature adipocytes, cathelicidin expression is significantly increased upon stimulation with the TLR1/2 agonistic lipopeptide Pam3Cys (*p* = 0.004) and the TLR2/6 agonistic lipopeptide MALP-2 (*p* = 0.004), respectively (Figure 3(c)). In parallel, TLR2 expression is strongly induced by both lipopeptides (*p* = 0.004) (Figure 3(d)). MALP-2 has more potent effects on cathelicidin and TLR2 expression when compared to Pam3Cys.

Identical stimulation experiments were repeated in undifferentiated 3T3-L1 fibroblasts. Cathelicidin expression (Figure 3(e)) and TLR2 expression (Figure 3(f)) are also highly inducible in fibroblasts. In these cells, effects of MALP-2 were not documented as superior to those of Pam3Cys.

### 3.9. Effect of Lipopeptide Stimulation on Adipocytic Secretion of Adipokines, Chemokines, and Cytokines into the Supernatant

Since TLR2 is expressed and upregulated during adipocyte differentiation and upon stimulation with lipopeptides, we studied the effect of lipopeptides on the secretion of adipokines (adiponectin, resistin, and lipocalin-2), chemokines (MCP-1), and cytokines (IL-6) into the supernatant of mature adipocytes and undifferentiated fibroblasts via ELISA (Table 3). In mature adipocytes, the secretion of the proinflammatory mediators MCP-1 and lipocalin-2 was significantly upregulated by each of the lipopeptides Pam3Cys and MALP-2, whereas the secretion of the anti-inflammatory mediator adiponectin was significantly downregulated upon stimulation with Pam3Cys. Both lipopeptides were not significantly affecting resistin secretion. In undifferentiated fibroblasts which normally exhibit a more proinflammatory potential lacking the expression of classical adipokines such as adiponectin, secretion of IL-6 and MCP-1 was analyzed. Basal IL-6 concentrations were generally very low. Pam3Cys and MALP-2 were able to stimulate MCP-1 and IL-6 secretion significantly.

## 4. Discussion

Systemic inflammation by circulating Gram-positive bacteria such as* Staphylococcus aureus* or their respective bacterial lipopeptides such as MALP-2 and Pam3Cys might affect adipose tissue biology at sites located distinctly from primary infection. Particularly, systemic inflammation together with the exposure to bacteria or bacterial lipopeptides might cause adipose tissue inflammation. During this process, activation of TLR2 and nucleotide-binding oligomerization domain protein-1 (NOD1) could represent a reasonable molecular mechanism capable of altering adipose tissue biology [49]. In the present study, we could demonstrate a shift in secretion profile from anti-inflammatory adipokines/cytokines such as adiponectin towards proinflammatory adipokines/cytokines such as visfatin, leptin, IL-6, PYCARD, and MCP-1. Gene expression profiles were similar between epididymal and subcutaneous adipose tissue depots in the mouse peritonitis model (PYCARD, IL-6, adiponectin, AdipoR1, visfatin, and LOX-1). Only for MCP-1, resistin, and leptin, we observed a differential gene expression profile. Whereas MCP-1 was upregulated in epididymal adipose tissue at all time points, its expression remained unchanged in subcutaneous adipose tissue. Resistin was only downregulated in epididymal adipose tissue but not in subcutaneous adipose tissue. Finally, leptin remained unchanged in subcutaneous adipose tissue but decreased in epididymal adipose tissue. PPAR*γ*, a transcriptional and specific regulator of adipocyte gene expression, also remained unchanged in subcutaneous adipose tissue but decreased in epididymal adipose tissue. Since epididymal adipose tissue in mice resembles visceral adipose tissue in humans, this site-specific difference in gene expression might be related to the pathophysiological process of peritonitis.

It is a matter of debate why functional toll-like receptors such as TLR2 are expressed in adipocytes. However, from an evolutionary point of view this pathway might coordinate energy utilization and distribution during severe infection and immune response. Moreover, the direct defense of adipose tissue against bacteria such as staphylococci might represent an additional and evolutionarily conserved mechanism of the innate immune system. The latter mechanism seems highly reasonable and meaningful since adipocytes and adipose tissue often reside at sites and tissue interfaces involved in complicated infections and diseases like bowel perforation (mesenteric adipose tissue), acute pancreatitis (retroperitoneal adipose tissue), chronic inflammatory bowel disease (mesenteric adipose tissue), arthritis (periarticular adipose tissue), ophthalmitis (retro-orbital adipose tissue), soft tissue-infection (subcutaneous adipose tissue), and many others [11]. Thus, the TLR2-cathelicidin pathway seems to be of major interest. Most importantly, we could demonstrate a strong induction of cathelicidin and TLR2 gene expression in vitro in differentiating adipocytes. Moreover, two of the major TLR2 agonistic lipopeptides, Pam3Cys and MALP-2, were shown to induce cathelicidin and TLR2 expression with high significance in both fibroblasts and mature adipocytes. These in vitro data strongly argue for a physiological significance of these evolutionary highly conserved signaling pathways and open new prospects for future research on adipocyte innate immunity. In contrast to our in vitro data, in the rodent models of* Staphylococcus aureus* infection used, we could not demonstrate a significant induction the TLR2 gene expression. However, TLR2 gene expression might be already at high levels in these mice and does not further increase during infection.

Transformation of white adipose tissue into beige or brown adipose tissue, a process recently named “browning” [50–53], might be triggered by systemic inflammation. However, we could exclude this possible mechanism by showing that UCP-1, one of the most prominent and highly specific markers of brown adipose tissue, was not induced in adipose tissue in endocarditis animals. Furthermore, other markers indicating “browning” such as TMEM26 and TBX1 were also negative.

Recent studies elucidated the role of epigenetic mechanisms in altered adipocyte phenotypical and functional programing during inflammation, involving regulation of adiponectin expression by modified promoter methylation [54]. These are intriguing novel aspects on adipocyte transdifferentiation processes and should be addressed by future investigation with respect to intra-abdominal and inguinal adipose tissue.

A deteriorated adipogenic differentiation program or redifferentiation into more premature adipocytes (fibroblasts) could possibly be caused by systemic inflammation. However, the white differentiation markers adiponectin, visfatin, and leptin were not changed in our experimental animals. Moreover, we could demonstrate via gene expression profiling that the main transcriptional inducer of white adipocyte differentiation, PPAR*γ*, was induced in epididymal adipose tissue in the murine peritonitis model whereas its expression levels were not changed significantly in the endocarditis model as well as in cultured adipocytes treated with TLR2-agonistic lipopeptides. Additionally, expression levels of common markers of lipid and carbohydrate metabolism in mature adipocytes such as PEPCK, Ptpn1, and LOX-1 were not downregulated but significantly upregulated. These data argue against a disturbed differentiation program during systemic inflammation in these animals.

Taken together, it is intriguing to speculate based on our data that adipocytes might be involved in the clearing of bacteremia and in the defense against systemic infection by* Staphylococcus aureus* [17]. This implication is meaningful with respect to the development of novel antibiotics and to the understanding of the pathophysiology of soft tissue-infections [17]. Importantly, stimulation by lipopeptides in vitro caused a significant and differential regulation of adipokines and cytokines in adipocytes on the basis of protein secretion into the supernatants. Both Pam3Cys and MALP-2 upregulated the secretion of proinflammatory proteins whereas the secretion of anti-inflammatory adiponectin was downregulated by Pam3Cys. These data strongly argue for the TLR2 pathway being both inducible and functional in adipocytes since effects were not only seen on the basis of gene expression but also seen on the basis of protein secretion.

Most in vivo studies have focused so far on either animal models with endotoxemia (lipopolysaccharide) or seriously diseased patients with sepsis that are presumably more heterogeneous. Only few studies are available using defined infection models with a single and well defined pathogen and only few studies investigated the effect of systemic infection on changes in adipose tissue itself directly. To our knowledge, no study has been published so far addressing the molecular effects of systemic infection by* S. aureus* on adipose tissue sites located distinctly from the infection site.

## 5. Summary

Following systemic bacterial infection by* Staphylococcus aureus*, there is a shift from secreted anti- to proinflammatory adipokines and cytokines in serum and on the level of gene expression in adipose tissue at sites distinct from infection. In contrast, the adipocyte differentiation program or the inducible mechanism of “beiging” or “browning” seems not to play a role in this context. The TLR2-cathelicidin pathway is expressed and inducible in murine 3T3-L1 adipocytes and fibroblasts in vitro. Classical lipopeptides are able to induce TLR2 and cathelicidin gene expression as well as the secretion of proinflammatory mediators from adipocytes.

## 6. Conclusions

In addition to the well-known process of metabolic inflammation (“adipoflammation”) in the context of insulin resistance, systemic infection by Gram-positive bacteria also induces proinflammatory transformation of adipose tissue. This effect is documented on the levels of gene expression and secreted mediators. The TLR2-cathelicidin pathway is expressed and highly inducible in adipocytes in vitro. Lipopeptides are important immune-modulators of adipocytes on the level of gene expression and protein secretion.

## Figures and Tables

**Table 1:** Gene expression levels in adipose tissue from *S. aureus* infected endocarditis animals.

Genes	Model	Adipose tissue	↑ ↓	Fold-expression ΔΔCt	*p*	*n*
*Inflammation*						
PYCARD	rat	epididymal	↑	1.17	0.0411	4
TLR2	rat	epididymal	↔	0.33	1.000	5–8
Lipocalin-2	rat	epididymal	↔	1.18	0.127	5–8
*Adipokines*						
Adiponectin	rat	epididymal	↔	−1.06	0.1903	4
AdipoR1	rat	epididymal	↓	−1.17	0.0398	4
Visfatin	rat	epididymal	↔	1.12	0.1503	4
Resistin	rat	epididymal	↑	1.80	0.0488	4
Leptin	rat	epididymal	↔	−0.88	0.073	5–8
*Browning*						
UCP-1	rat	epididymal	n.d.			
CD137	rat	epididymal	n.d.			
TBX1	rat	epididymal	n.d.			
TMEM26	rat	epididymal	n.d.			
*Lipids/metabolism*						
Ptpn1	rat	epididymal	↑	5.14	<0.001	4
PPAR*γ*	rat	epididymal	↔	−2.72	0.1322	4
PEPCK-1	rat	epididymal	↓	−0.68	0.0202	4

Gene expression in epididymal adipose tissue from rats with endocarditis was analyzed. This table depicts the gene expression difference (ΔΔCt) versus uninfected control animals for genes with a ΔΔCt of at least +/−1. PYCARD, PYD and CARD domain-containing; TLR2, toll-like receptor-2; AdipoR1, adiponectin receptor type 1; Ptpn1, protein tyrosine phosphatase-1; PPAR*γ*, peroxisome proliferator-activated receptor-*γ*; PEPCK-1, phosphoenolpyruvate carboxykinase; UCP-1, uncoupling protein-1; TBX1, T-box 1; TMEM26, transmembrane protein-26.

↔: no change; ↑: upregulation; ↓: downregulation.

**Table 2:** Gene expression levels in adipose tissue from *S. aureus* infected peritonitis animals.

Genes	Model	Adipose tissue	↑ ↓	Fold-expression ΔΔCt	*p*	*n*
*Inflammation*						
PYCARD	Mouse, 24 h	Epididymal	↑	1.83	0.0002	6
PYCARD	Mouse, 48 h	Epididymal	↑	2.25	0.0001	5
PYCARD	Mouse, 24 h	Subcut.	↑	0.76	0.0183	6
PYCARD	Mouse, 48 h	Subcut.	↑	0.96	0.0434	5
Interleukin-6	Mouse, 24 h	Epididymal	↑	4.23	0.0025	6
Interleukin-6	Mouse, 48 h	Epididymal	↑	7.90	0.0001	5
Interleukin-6	Mouse, 24 h	Subcut.	↑	2.35	0.0310	6
Interleukin-6	Mouse, 48 h	Subcut.	↑	1.92	0.0118	5
MCP-1	Mouse, 24 h	Epididymal	↑	3.23	0.0003	6
MCP-1	Mouse, 48 h	Epididymal	↑	4.68	0.0001	5
MCP-1	Mouse, 24 h	Subcut.	↔	2.43	0.0893	6
MCP-1	Mouse, 48 h	Subcut.	↔	1.22	0.1340	5
TLR2	Mouse, 24 h	Epididymal	↔	0.94	0.054	7-8
TLR2	Mouse, 48 h	Epididymal	↔	0.77	0.315	4–7
TLR2	Mouse, 24 h	Subcut.	↔	−0.23	0.686	4
Cathelicidin	Mouse, 24 h	Epididymal	↔	−0.30	0.798	8
Cathelicidin	Mouse, 48 h	Epididymal	↔	−0.15	0.933	4–8
Cathelicidin	Mouse, 24 h	Subcut.	↔	1.98	0.343	4
Lipocalin-2	Mouse, 24 h	Epididymal	↑	6.84	0.001	6–8
Lipocalin-2	Mouse, 48 h	Epididymal	↑	6.05	0.010	4–6
Lipocalin-2	Mouse, 24 h	Subcut.	↑	5.14	0.029	4
*Adipokines*						
Adiponectin	Mouse, 24 h	Epididymal	↔	−0.97	0.2870	6
Adiponectin	Mouse, 48 h	Epididymal	↔	−0.51	0.4830	5
AdipoR1	Mouse, 24 h	Epididymal	↔	−0.40	0.2193	6
AdipoR1	Mouse, 48 h	Epididymal	↔	−0.36	0.3466	5
Visfatin	Mouse, 24 h	Epididymal	↔	−0.20	0.7833	6
Visfatin	Mouse, 48 h	Epididymal	↔	−0.22	0.7944	5
Resistin	Mouse, 24 h	Epididymal	↓	−1.73	0.0166	6
Resistin	Mouse, 48 h	Epididymal	↓	−1.52	0.0451	5
Resistin	Mouse, 24 h	Subcut.	↔	0.01	0.9895	6
Resistin	Mouse, 48 h	Subcut.	↔	−0.56	0.0688	5
Leptin	Mouse, 24 h	Epididymal	↓	−1.87	0.007	15–17
Leptin	Mouse, 48 h	Epididymal	↓	−2.94	0.005	13–15
Leptin	Mouse, 24 h	Subcut.	↔	−2.04	0.057	4
*Browning*						
CD137	Mouse, 24 h	Epididymal	↔	1.61	0.050	8
CD137	Mouse, 48 h	Epididymal	↑	2.17	0.004	4–8
CD137	Mouse, 24 h	Subcut.	↔	0.51	0.886	4
UCP-1	Mouse, 24 h	Epididymal	↔	−0.07	0.886	4
UCP-1	Mouse, 24 h	Subcut.	↔	6.70	0.114	4
*Lipids/metabolism*						
LOX-1	Mouse, 24 h	Epididymal	↑	2.91	0.0023	6
LOX-1	Mouse, 48 h	Epididymal	↑	5.58	0.0004	5
LOX-1	Mouse, 24 h	Subcut.	↑	3.03	0.0110	6
LOX-1	Mouse, 48 h	Subcut.	↑	2.39	0.0002	5
PPAR*γ*	Mouse, 24 h	Epididymal	↓	−0.55	0.035	17-18
PPAR*γ*	Mouse, 48 h	Epididymal	↓	−1.72	<0.001	13–17
PPAR*γ*	Mouse, 24 h	Subcut.	↔	−0.38	0.343	4

The expression of selected genes in epididymal and subcutaneous adipose tissue obtained from mice with S*. aureus* peritonitis was analyzed and compared to adipose tissue from uninfected control animals. This table depicts the gene expression difference (ΔΔCt) versus uninfected control animals for genes with a ΔΔCt of at least +/−1. MCP-1, monocyte chemoattractant protein-1; PYCARD, PYD and CARD domain-containing; AdipoR1, adiponectin receptor type-1; CD137, tumor necrosis factor receptor superfamily, member 9; LOX-1, lectin-like oxidized-LDL receptor-1; Ptpn-1, protein tyrosine phosphatase-1; UCP-1, uncoupling protein-1; PPAR*γ*, peroxisome proliferator-activated receptor-*γ*; subcut., subcutaneous adipose tissue.

↔: no change; ↑: upregulation; ↓: downregulation.

**Table:** (a) 3T3-L1 adipocytes

	Ctrl.	50 ng/ml Pam3Cys	100 ng/ml Pam3Cys

MCP-1 [ng/ml]	0.082 ± 0.040	1.182 ± 0.058^*∗∗*^	1.260 ± 0.230^*∗∗*^
Adiponectin [*µ*g/ml]	109.91 ± 16.92	98.55 ± 13.85	79.58 ± 16.62^*∗*^
Lipocalin-2 [*µ*g/ml]	9.89 ± 2.34	15.54 ± 3.11^*∗∗*^	13.83 ± 2.68
Resistin [*µ*g/ml]	28.08 ± 4.92	29.67 ± 5.96	27.66 ± 8.18

	Ctrl.	50 ng/ml MALP-2	100 ng/ml MALP-2

MCP-1 [ng/ml]	0.216 ± 0.045	3.743 ± 0.564^*∗∗*^	3.853 ± 0.433^*∗∗*^
Adiponectin [*µ*g/ml]	70.31 ± 8.14	65.18 ± 11.40	59.60 ± 6.40
Lipocalin-2 [*µ*g/ml]	5.72 ± 1.03	15.81 ± 1.65^*∗∗*^	16.76 ± 3.24^*∗∗*^
Resistin [*µ*g/ml]	10.99 ± 0.93	9.35 ± 1.33	9.31 ± 1.63

**Table:** (b) 3T3-L1 fibroblasts

	Ctrl.	50 ng/ml Pam3Cys	100 ng/ml Pam3Cys

MCP-1 [ng/ml]	1.794 ± 0.182	6.192 ± 0.752^*∗∗*^	5.550 ± 0.477^*∗∗*^
IL-6 [pg/ml]	40.61 ± 4.99	226.61 ± 39.80^*∗∗*^	207.97 ± 26.27^*∗∗*^

	Ctrl.	50 ng/ml MALP-2	100 ng/ml MALP-2

MCP-1 [ng/ml]	0.875 ± 0.095	6.803 ± 1.495^*∗∗*^	6.956 ± 1.492^*∗∗*^
IL-6 [pg/ml]	32.80 ± 10.57	98.40 ± 26.28^*∗∗*^	78.59 ± 25.50^*∗∗*^

Mean concentrations ± double standard error of the mean (2x SEM) are summarized by using 4–6 wells each. Concentrations were normalized to total cell protein. MCP-1, monocyte chemoattractant protein-1; ^*∗*^*p* < 0.05 and ^*∗∗*^*p* < 0.01.
